# Challenges and opportunities confronting female-headed households in Iran: a qualitative study

**DOI:** 10.1186/s12905-020-01046-x

**Published:** 2020-08-17

**Authors:** Javad Yoosefi Lebni, Mohammad Ali Mohammadi Gharehghani, Goli Soofizad, Bahar khosravi, Arash ziapour, Seyed Fahim Irandoost

**Affiliations:** 1 Health Promotion Research Center, Iran University of Medical Sciences, Tehran, Iran; 2 Social Welfare Management Research Center, University of Social Welfare and Rehabilitation Sciences, Tehran, Iran; 3 School of public Health and Safety, Shahid Beheshti University of Medical Sciences, Tehran, Iran; 4 Students Research Committee, Kermanshah University of Medical Sciences, Kermanshah, Iran; 5 Health Education and Health Promotion, Health Institute, Kermanshah University of Medical Sciences, Kermanshah, Iran; 6 Health Education and Health Promotion, Department of Public Health, School of Health, Urmia University of Medical Sciences, Urmia, Iran

**Keywords:** Female-headed households, Challenges, Opportunities, Qualitative content analysis, Iran

## Abstract

**Background:**

Female-headed households are one of the most vulnerable groups of society that confront many problems and challenges. Therefore, the present study aimed to explore the challenges and opportunities confronting female-headed households in Iran.

**Methods:**

This qualitative study was conducted among female-headed households in Kermanshah, West of Iran, in 2019. The data were collected through Semi-structured interviews with 26 female-headed households who were selected by purposeful and theoretical sampling. Data analysis was done through conventional qualitative content analysis, and the software MAXQDA-12 was used for the management of data. The four criteria of Goba and Lincon, including credibility, confirmability, dependability, and transferability, were observed to evaluate the quality of research results.

**Results:**

After analyzing the data, 4 main categories and 13 subcategories were obtained as follows: individual problems (role overload, role conflict, end of love, psychological problems), intra-family problems (declined independence, intra-family tension, poverty reproduction and family disability), social problems (stigma of being unattended, social insecurity, social isolation, social exclusion), positive outcomes (positive self-concept, social maturity).

**Conclusion:**

Female-headed households face many challenges that can become a big threat or an opportunity. Therefore, their health improvement can be achieved through training and helping them to adapt to new and multifaceted roles, providing more economic support and helping them raise their social status.

## Background

Development and social change have led to changes in family structure [1]. One of these changes is the formation of single-head or single-parent families [1–3]. The head of the household is usually responsible for all or most of the household expenses or deciding how to spend the household income and is not necessarily the oldest member of the household and may be male or female [4]. A female head of household refers to a woman in charge of managing the family as a result of divorce, separation, immigration, or widowhood [5].

The number of female-headed households has increased dramatically in the recent half-century, especially in developing countries [6], due to divorce, spouse death, addiction or disability of husband, increased life expectancy among women, migration, or being abandoned by husband [7, 8]. According to worldwide statistics, the rates of female-headed households in different countries vary. In 2007, 49.40% of households in Ukraine had female heads. In Namibia in 2013 the rate was 43.90% and in 2015 in Zimbabwe 40.60% of households had female heads. In 2016, Armenia had the highest rate of female-headed households with 33.20% [9]. In Iran, there is a growing number of formal single-parent households headed by women [10]. In 2006, the proportion of female-headed households was 9.5%, in 2011 the proportion was 12.1%, and it reached 12.7% in 2016 when there were 3,061,753 female-headed households in Iran [4].

There is a general perception that women are socially more vulnerable than men because of higher poverty rates and fewer job opportunities, and this perception is more widespread for female-headed households due to fears of intergenerational poverty transmission [11, 12]. Female-headed households are forced to play multiple, conflicting roles after losing their spouses, and have to work in marginal, part-time, informal, and low-income jobs due to lack of access to high-paying jobs [13]. These women are unable to maintain their health due to problems such as poverty, poor socioeconomic status and multiple responsibilities [3, 14–16]. As a result, they experience more high-risk behaviors [15, 17] and lower quality of life and family satisfaction [15–17]. They also suffer from Stress, mental disorders, depression, drug abuse, and financial and cultural poverty [16, 18, 19]. The results of the Rezaei et al., 2013 study showed that female-headed households are highly vulnerable and experience many problems such as low income, widespread economic problems, mental, neurological and physical disorders and isolation [1]. Veisani et al., (2013) also showed that female-headed households have poor health and quality of life, and the most critical factors associated with this low quality of life are low literacy and chronic diseases [20].

Women are among the most prestigious groups of society because they form the foundation of family and society’s health [6, 21]. Providing them with the care they need is possible when there would be a broad and comprehensive view of their situation and problems [22]. Most studies on female-headed households have been quantitative and empirical, focusing on their economic problems [1, 20, 23]. However, to better understand the challenges and problems of female-headed households, there is a need for qualitative research that covers all aspects of their lives and shows their living conditions from the perspective of their own experiences and views. Therefore, the purpose of this study was to identify the challenges and opportunities facing female-headed households in Iran with a qualitative approach.

## Methods

### Design

A qualitative method with a conventional content analysis approach was used to conduct the present study. The qualitative method emphasizes the deep understanding, complexity, and details of the phenomena under study and the researcher is actively involved in the research process. In conventional content analysis, most data is obtained through interviews, and interviews with individuals allow them to understand, participants’ experiences and perceptions and gain richer data from their experiences [24–27].

### Setting and participants

The present study was carried out in Kermanshah province in western Iran in 2019. According to the official statistics of Iran in 2016, there were 75,788 female-headed households in this province [4]. Participants in the study included 26 female-headed households whose spouses had died. Inclusion criteria included being the head of the household due to the spouse’s death, having children, and being willing to participate in the study.

### Data collection

Data collection started after receiving the code of ethics from Kermanshah University of Medical Sciences and entering the research field. Data collection lasted 4 months from June to September of 2019. First, purposeful sampling was used to select the interviewees. Then theoretical sampling was used to identify the characteristics of the individuals and to find the path of research. This type of sampling with a variety of information allows for a better study and analysis of the nature and dimensions of the phenomenon [26]. To achieve maximum variation in data, it was attempted to select samples from female-headed households with different economic and social characteristics (such as age, level of education, occupation, etc.).

A semi-structured interview was used to collect data. That is, the researcher first began by asking demographic questions and then provided the ground for more in-depth discussions by asking general questions. The interviews were carry out by the first author together with the third authors, who had a previous experience in qualitative research and semi-structured interviews and also had previously conducted qualitative studies in the same areas. Some of the questions were as follows: What problems did you have after becoming the head of household? What changes happened in your life? What changes did you experience in your role as a woman? What changes happened in your family after the death of your husband? What was your most important problem in managing your family? What was the attitude of your associates toward you and how did they treat you? What were the most critical problems you had in society, explain?

The criterion for determining the number of samples was theoretical saturation which was obtained by interviewing 26 female-headed households in Kermanshah province. In qualitative research, theoretical saturation is when the continuation of the interviews no longer helps to generate new data and all codes are repeated so the researcher decides to stop the interview process [28]. In this study, all codes were repeated after interview 21, but the researchers continued the interview process for up to 26 people for more accuracy and confidence that they avoided false saturation. After getting sure that no new code was generated, the researchers did not continue the interviews. Only two interviews were postponed to the participants’ request to another time which was later conducted by the researchers; no other participant withdrew during the interviews. Each interview took an average of 60 min. The highest and lowest intervals were 36 and 80 min, respectively. The interviews were conducted in the selected participants’ private homes and a secluded place without anyone else. The favorable conditions remain such that the participant could willingly participate in the interview without any worries and swiftly respond questions while maintaining her privacy.

In order to observe the research ethics at the beginning of each interview, participants were informed about the purpose of the study and the optionality to participate in the study. Also written consent was obtained from all participants and a parent or guardian on behalf of any participants under the age of 18 to record the interview. We did not have any participant under 16 years. They were assured that their personal information would remain confidential and at the time of publication of results, their names and addresses would not be published. In some cases, when they wanted a female interviewer, a trained female researcher was used to conduct the interview.

### Data analysis

Data analysis was performed simultaneously with data collection based on the method of Graneheim and Lundman [29]. Thus, immediately after each interview, the researcher and their colleague listened to the content of the interview twice and then reviewed the text several times to allow the researcher to get a general sense of the text. At that point, to manage data, the conversation was transcribed and entered into the MAXQDA-12 software. The data were followed word by word, and the first level of coding process was started by identifying and highlighting the sentences and paragraphs of the unit of analysis with emphasis on explicit and implicit content. Each unit was then given a code, and the categories were extracted. Then the codes were reduced to categories based on differences and similarities.

### Trustworthiness

To ensure quality of the results, the Lincoln and Guba criteria were applied [26, 30, 31]. The researchers were natives of the study areas, and one of them had a 7-year experience working in centers for female-headed households, so this created a sense of trust in the participants and could express their experiences more easily (credibility). In the data analysis process and coding, the needed help received from two academic experts and researchers familiar with qualitative research (sociologist and health promotion specialist) two social workers who actively engaged organizations related to female-headed households and one woman specialist (Master of Women’s Studies) who had done the investigation and practical works in the similar field. In the end, the results were also provided to 7 samples to determine whether the results reflected their experiences and living conditions well or not (dependability). The researchers throughout the research process attempted to put aside their personal views and record all aspects and observations and not to entail their presupposition in data collection and analysis as much as possible. Since the process of coding and data analysis was done in groups and the entire research team, individuals could not interpret their personal biases and the study results (Confirmability). A description of the categories extracted was provided with those who did not participate in the study and an agreement on categories was reached with them. Also individuals with different socioeconomic status, educational status, and different ages were selected (transferability).

## Results

The present study was conducted with the participation of 26 female-headed households in Kermanshah province. Findings showed that most of the sample lived in the age range of 20–30 years, in rural areas and families with three persons and were under diploma and housekeeper (Table 1).

**Table 1:** Demographic characteristics of the participants

Characteristic	Frequency (*n* = 26)	%
Age		
< 19	5	19.2
20–30	10	38.5
30–40	6	23.1
> 40	5	19.2
Level of education		
Illiterate	6	23.1
Under diploma	11	42.3
Diploma and above diploma	9	34.6
Employment status		
Housewife	15	57.7
Self-employed	9	34.7
Employee	2	7.6
Residence		
City	11	42.3
Village	15	57.7
Household size		
Under 3 people	15	57.7
4–5 people	5	19.2
more than 5	6	23.1
Average time of being household head		
Under 10 years	10	38.5
10–20 years	12	46.1
Above 20 years	4	15.4

After analyzing the data, four main categories and 13 sub-categories were identified (Table 2), which are examined separately.

**Table 2:** Categories and sub-categories

Categories	Sub-categories
Individual problems	Role overload
	Role conflict
	End of love
	Psychological problems
Intra-family problems	Declined independence
	Intra-family tension
	Reproduction of Poverty and Family Disability
Social problems	stigma of being unattended
	Social insecurity
	Social isolation
	Social exclusion
positive consequences	Positive self-concept
	Social maturity

### Personal problems

The first category of research addresses the individual problems that women experience when they become household heads. These problems include role overload, role conflict, psychological problems, and the end of love.

- **Role overload**

Although a woman plays the head of the household, these women must simultaneously play roles in addition to the mother’s role, which causes them to face challenges. Female-headed households simultaneously fulfill all the roles of parents that subsequently, they have to bear a great deal of pressure, in many cases beyond their capacity. *“It makes me tired that I have to do the housework and work outside of the home at the same time. Most of the time, I do not have time to rest at all.” (24- year-old housewife)*

*"During the days I go to work, and when I return home in the evening, I have to do the housework. When I finish the housework, getting tired and exhausted, I have to prepare food for the kids' tomorrow lunch, most of the time I don't know when I sleep" (35- year-old employee).*

Taking on multiple roles enforces female-headed households to work all day long and puts enormous pressure on them, which can threaten their physical and mental health during a long period and cause them to face physical and mental depreciation.

- **Role conflict**

Playing different roles by female-headed households, especially the role of parents causes conflict because, in many cases, these roles conflict according to customs and roles defined for men and women in their community.

*"Sometimes, I don't know how to treat kids like a kind mother or a powerful father. If I just play the role of a mother, my kids may not listen to me anymore, or if I'm too strong, I'm afraid my kids won't be comfortable anymore." (45- year-old housewife)*

*"When a suitor proposes to my daughter I didn't know what role I should have, I had a terrible feeling. It was tough to play two roles of parents at the same time” (50- year-old housewife)*

Therefore, taking on multiple roles, which sometimes conflict with reasons of social customs stemming from a patriarchal system in the areas under study, can put a great deal of pressure on women and force them to accept multiple and conflicting roles.

- **End of love**

After the death of the husband, the female-headed households do not have an excellent chance to get married and if they have a suitor, they must choose based on their circumstances – the number of children, financial status, etc. Also, in the field of study, due to cultural measures, women are sometimes forced to marry their brother-in-law after the death of their husbands, and with forced marriage, love is almost over for them.

*"My husband died, a suitor came for me a couple of years after his death, but I couldn't give him a positive answer, I liked to get married, but I knew he couldn't accept my children, so I don't think about marriage anymore" (28- year-old self-employed)*

*"After my husband died, my feeling died too, because I knew I didn't have the right to fall in love. If I wanted to get married, I would just have to marry someone who would accept my condition that I usually marry a man who is 20 or 30 years older than me." (21- year-old housewife)*

*"After the death of my husband, his family forced me to marry my brother-in-law. He was a few years younger than me, and we did not like each other at all, but they forced two of us to accept this marriage, we couldn't put up with each other, and he left me a few months later." (33- year-old housewife)*

In fact, women in the areas under study after the death of their husbands are considered the husband’s property, which must be owned by the husband’s family. Thus after the husband’s death, the wife is forced to marry a male member of his family. Otherwise, few men agree to marry female-headed households due to the views about the case.

- **Mental health problems**

After the death of the husband, the female-headed household, suffers sever living conditions and role conflicts, endure stress and mental pressure that lead to mental illnesses and traumas such as depression, self-immolation, and Lack of happiness and hope for life and the future.

*"After my husband’s death, I got into so much financial and nonfinancial trouble that I went to the limit of insanity. I often feel I’m depressed. Nothing makes me happy" (17- year-old housewife)*

*"After my husband's death, so many problems came up that I got a mental illness, I was hospitalized for a while, and I take pills. It’s really tough to be both father and mother alone." (18- year-old self-employed)*

*"After my husband's death, I had so many problems and was mentally and socially in distress that I committed self-immolation, but unfortunately, I didn't have the chance and didn't die." (28- year-old housewife)*

As a matter of fact, the social and economic pressures exerted on female-headed households expose them to many mental illnesses that they may find no way except suicide at the end.

### Intra-family problems

The second category that female-headed households experience and deal with its problems is problems and challenges within the family, including sub-categories of declined independence, intra-family tension, and the reproduction of poverty and family disability.

- **Declined independence**

In Iranian society, especially in the area of study, when a woman is in charge of the household, the family of her husband and her own family come closer to her to support her, especially if the woman has a child, and this may lead to interference in woman’s life and decisions and affect the independence of her family.

*"Ever since my husband left [this world], his family has interfered in my life, and my children live more than ever before, and sometimes I have to have their agreement to make important decisions or otherwise they won't let me do it." (45- year-old housewife)*

*"My daughter’s suitor is a good boy. He is accepted by my daughter and me totally, but my husband's family disagrees, and they don't allow this marriage to happen, thinking they should interfere in everything in our lives" (46- year-old housewife)*

After the death of the husband, since there is no confidence to manage the family, the family of the woman and especially her husband’s family, supposing they are supporting, begin to intervene and make decisions for the woman and her children and disrupt their personal and private lives so that they even lose the opportunity to make decisions in their most personal matters.

### Intra-family tension

When a woman becomes head of a household, many family members may not be able to cope with the new role of mother as family head and manager for a long time. Children disobey his orders, challenge each other, and even consider them independent. This process creates tension between the family and the mother.

*"When my husband died, I tried very hard that fatherlessness does not bother my children, but the kids, especially my older son, competed with me, thinking he should take over family management. In fact he didn't accept me as the head of the family. This view caused much tension between us.” (33- year-old self-employed)*

*"After my husband's death, I could not control my children. Each of them went their way, and none of them accepted me as the head of the household" (35- year-old housewife)*

Due to the gender stereotypes and the patriarchal system in the area under study, accepting a woman as head of household can be difficult even for male children, and there can be resistance, so women have their intra-family pressures in addition to social pressures. In the family, they face some rejection.

- **The reproduction of poverty and family disability**

Since most women in the study area have low literacy and are unable to pursue a specific occupation, they face many problems after their husband’s death and accepting the head of the household role. In some cases, children from these families are forced to work as child workers and stay away from school. Somehow the cycle of poverty within the family is reproduced, and poverty is passed on to the next generation, with an indefinite future awaiting them.

*"My husband was a builder. When he died we had no income except the money we received from the Subsidy (a financial help by the government) and the Relief Foundation, I didn't know what to do, so my sons were forced to leave school and go to work as a laborer" (50- year-old housewife)*

*"Since I was alone we haven’t had any mentionable income, the conditions of the community were such that I could not go out for work, so I had to send my children to work (as a laborer), I get very annoyed because I know I am ruining their future, but I have no choice." (55- year-old housewife)*

In Iran, women are not provided with proper economic and social support, and the socio-cultural conditions do not allow them to work outside the home in many cases, so the economic burden of these families is more on the children. Thus many children are forced to start working at an early age, drop out of school and no longer have a chance to have a better life in the future, resulting in the reproduction of poverty and disability in the female-headed family.

### Social problems

The third category that female-headed households are engaged in is social problems, which include sub-categories of the stigma of being unattended, social insecurity, social isolation, and social exclusion.

- **Stigma of idleness**

Households whose men (husbands) were absent from the family have long been called unattended, and this label’s use has continued to this day. Although it may not be socially harmful, it often puts a lot of stress and pressure on female-headed households and discourages them from continuing with their lives.

*"I get bothered that they consider my family unattended all the time. They talk in a way as if I'm not a human; when they say like this I feel they don't see the things I do for my kids" (38- year-old housewife)*

*"When people call me “unattended”, I feel bad. I feel weak. I hate this word." (30- year-old self-employed)*

Since there are still many gender stereotypes in the community under study, considering a woman as head of household is unacceptable to many, so people consider female-headed households unattended, and there is the stigma of being unattended.

- **Social insecurity**

Female-headed households are identified as vulnerable groups that may be abused and subjected to violence by society and associates and lack the security to live and maintain their families.

*"When people see that I am alone and do not have anyone with me, they allow themselves to make any impudent offers. Many times even those close relatives offer me sex." (28- year-old employee)*

*"Most of the time, when I go out, I don't tell strangers that I don't have a husband. I often have to tell a lie and say I have a husband because I know that if they find out I don't have a husband, they will want to get close to me in any way and hurt me" (33- year-old self-employed)*

Female-headed households are a vulnerable and defenseless group of the community and they may be harassed at times in the community, and not able to appear in society quickly. So, as a result, they confront some social insecurity.

- **Social isolation**

Women heads of households are more in the public eye and more at the center of attention than other women. That is why they may have to appear less in the community due to fear of being defamed.

*"I have to go out less and talk to men less because I fear people talk behind me." (26- year-old housewife)*

*"Before my husband’s death, I could go out shopping alone and easily. I could go to family and friends parties and do a lot of other things, nobody said anything, but since my husband died, I have not been as comfortable as before because I know the people talk behind me. If they see me talking to a shopkeeper or going out a lot, they think badly about me." (29- year-old self-employed)*

In fact, since the community does not have a positive view on female-headed households and they are in the public eye and under stigmatization, these women prefer to choose a form of isolation and seclusion to maintain their privacy and social status.

- **Social exclusion**

Female-headed households experience unkindness in society and are marginalized by society because of the way they are viewed as widows. These women are not accepted by society and are rejected as people who do not have social norms.

*"When I want to rent a house, it’s tough for me to have a house just because I don't have a husband" (33- year-old self-employed)*

*"Many families do not want us to be in touch with their wives or sisters. Many times they do not allow them to come to our home. One of my old friends who was in touch with me all the time cut contact with me after my husband died. Then I realized that her husband had not let her come to me. "(45- year-old housewife)*

The loss of a husband creates a negative view of women among her associates affecting her social relationships. Many people avoid having relationships with female-headed households because they do not want to be exposed to stigma. As a result, female-headed households are excluded from society.

### Positive consequences

The last category that female-headed household experience is the desirable consequences that result from being the head of the household. This category includes the sub-categories of social maturity and positive self-concept.

- **Positive self-concept**

Being head of a household can have a positive side, too. When some female-headed households try to find a job after becoming in charge of the household and find a job they take a more positive view of themselves and their abilities .

*"I used to stay home until my husband was alive, but when he died, I had to get a driving license and work as an agency driver. I feel good. I feel so powerful that I can do anything." (33- year-old self-employed)*

*"After my husband died, I decided not to let my kids feel any lack in life. I rented a shop and started making and selling local dairy. Thank God, my income is good. I was anxious early, but now I feel really good about myself. I feel that my self-confidence has increased a lot." (40- year-old self-employed)*

Since women are forced to run families on their own after their husbands’ deaths and have a job, this leads to a sense of positive self-concept in women.

- **Social maturity**

Women in the study areas are less likely to appear in the society due to limitations and social and cultural conditions, but after the death of their husbands, conditions may be prepared for them to come more in the society and pursue social and occupational activities. They may be successful.

*"Before my husband died, I used to go out rarely, and he did all the works of outside, but since he was gone, I've been doing it myself. I go out more and deal with many people. I've been setting up a community for a while. "(35- year-old housewife)*

*"Two years after my husband died I took part in the village council’s elections, and I was elected. I never thought I'd do it someday, but after my husband's death, when I was in touch with people more, I realized I could be a successful person." (28- year-old employee)*

As women are forced to move beyond the family and appear in society after the husband’s death, this results in broader relationships and better work conditions that lead to a form of social maturity in these women.

## Discussion

The present qualitative research aimed to identify the challenges and opportunities facing female-headed households in Iran. The result showed that these women encounter many selves, family, and social problems that can endanger their health. More result confirm the issue of female-headed households inevitably was not an obstacle and a barrier to women, but also, in some cases, it can enhance an opportunity and improve self-esteem and social maturity.

The first category that female-headed households confront is individual problems and challenges. Due to the nature and status of their roles, female-headed households have multiple tasks that often lead to many problems and they experience role conflicts and inability to play roles. Various studies have shown that changing the family structure from two parents to single parents presents many challenges for each person [32, 33]. In Habib’s (2017) research, women complained about the massive role of head of household, the effort to earn a living, and a large amount of activity leading to fatigue, physical injury, and disability [34]. Herbst (2012) also cited multiple tasks as one of the significant challenges for women heads of households [35]. Given that women in Iran and the regions under study become sociable in accepting feminine and domestic roles and lack the preparedness and experience of household management, they are naturally challenged to perform the household head’s new role. The multiplicity of roles and masculine duties alongside feminine duties make them desperate. Under these circumstances, the lack of women’s support institutions, lack of adequate support for women, and lack of education aggravate the role conflict and the difficulty of performing household duties.

Another challenge that female-headed households face is the low chance of remarriage or forced marriage and the end of love after their husband’s death, which sometimes leads to marriage with brother-in-law. Like our results, Yoshida’s (2011) study showed that women are less likely to marry after losing their spouses for cultural reasons [36]. The cultural and social characteristics of Kurdish areas, such as the tendency of men to marry virgin daughters or to reject women with children from previous marriages, make female-headed households limited to remarry and often lack a willing and loving marriage. Moreover, just due to society’s view that women must have a man as head of household, they accept the marriage and couples often have a significant age difference. Also, patriarchy, jealousy, blood, and cultural prejudice cause the family of the deceased husband to make her marry her brother-in-law, often leading to separation and divorce or emotional divorce.

Another finding of this study was the existence of mental problems in female-headed households consistent with previous studies in this area [37, 38]. Other studies have also referred to the psychological problems and depression of female-headed households [39, 40]. In a study conducted by Yoosefi Lebni et al., (2019) and Mirzaee et al. (2015) in the Kurdish areas of Iran, female-headed households as a vulnerable group committed self-immolation [41, 42]. Many socio-economic pressures and the lack of supportive organizations cause many problems for the female-headed household, which leads to many psychological problems.

One of the exciting results of this study, which is less discussed in previous research, is the occurrence of intra-family problems in female-headed households. The family of the deceased husband restricts the independence of female-headed households. In Kurdish areas, and a broader view in Muslim communities, issues of generation, paternity, and children belonging to the father’s family make women less empowered to decide on family matters, especially the future of their children, and the family of the father-in-law think they have full authority to interfere. In most cases, this situation leads to a challenge between the woman head of the household, and her husband’s family and children. The number of people willing to decision-making makes it challenging to reach an agreement and makes women helpless.

Female-headed households in this study confronted a lot of family tension with children and a lack of control over children. In the Arends-Kuenning and Duryea (2006) study, there were tensions and challenges between female-headed households and children [43]. The difficulty of a woman headed household roles, especially in a masculine position, causes both the woman to be incapable of performing her duties and the children to disobey her. This challenge also goes back to cultural beliefs and patriarchal dominance. According to sociability, authority and guardianship are for men. That is why children, especially male children, do not follow the mother and many challenges arise.

Another interesting finding in this study, which has been less addressed in previous research, was the reproduction of poverty and disability in female-headed households. In female-headed households, due to the lack of male breadwinners, children go to work instead of education, and their future is tied to poverty. In the literature on female-headed households and poverty, female-headed households are regarded as the “poorest of the poor” [44] and there exists a phenomenon of the feminization of poverty; the spread of poor female-headed household [45]. Numerous studies have shown a link between female-headed households with poverty [5, 46–48] and low socioeconomic status [44]. Arias and Palloni (1999) also found that children raised in female-headed households suffer from negative social and economic impacts throughout adulthood and have lower educational and career advancements [49]. Since women in society do not have the same breadwinner status as men and often work at lower levels, they naturally receive lower wages and experience greater poverty. This gender inequality causes sons to pursue income, resulting in the reproduction of poverty and failure to reach a high economic and social status.

In addition to individual and family challenges, female-headed households faced social problems. One of the issues that bother female-headed households is the label of being unattended for female-headed households, consistent with Towers’s (2005) research [50]. In the Habib (2017) study in Bangladesh, women heads of households also received social stigma, and society did not view them positively [34]. Social and cultural norms and doctrines have been developed for men’s household management, and society reacts when women are in charge because they do not accept their position, which leads to stigmatization by society.

The formation of a sense of social insecurity in female-headed households was another finding of this study that is consistent with previous research [34]. The lack of men and the loneliness of women allow people, especially men, to exploit them sexually and even asexually (financial abuse, psychological harm, and so on.) and make them vulnerable in general.

They also experience some form of social exclusion in line with the social insecurity with which women are involved. In a study by Thomas and Ryan (2008), the results showed that losing the husband causes the loss of the significant support, and many women lost the support and companionship of their friends after they were left alone by their husbands [51]. Other studies have also shown that being the head of household and lacking men leads to loss of opportunities and social support from families and relatives [34, 52]. Since women heads of households are alone and do not have someone to meet their sexual needs, they are always viewed by society as potentially diverting or having the potential to deviate. Therefore many people fear getting close to them even if they do not have bad intentions because they may be labeled in society. As a result, after their husband’s death, the female-headed households would be in a narrower social circle and somehow rejected.

Female-headed households in our study stated that when they experience social exclusion and insecurity, they choose a form of isolation and prefer to be less present in society to avoid stigma. In the study of Finkelstein, 2011, similar to our findings, women opted out of social activities and chose seclusion [53]. Inappropriate social attitudes to female-headed households and harassing them to lead to these women being marginalized and confined to a limited circle of relationships with their family members and losing many of their social opportunities.

While previous research has focused more on revealing the negative consequences of becoming a household head, the results of this study reveal that household leadership for women is not only with negative consequences but also with positive outcomes, such as positive self-concept and self-efficacy, and social maturity. This concept was one of the exciting new findings in this study.

Since female-headed households have to try for better conditions and support for the family, they feel powerful in managing life and believe that they are capable of progress and success. These conditions lead to social maturity and the expansion of women’s participation in outdoor activities. Although this experience has not been found for female-headed households in other studies, some research has shown that after divorce, women are given new opportunities for emotional and individual development, a sense of freedom, and experience of new challenges [51, 54]. In fact, after their husband’s death, women are forced to take on some outdoor works and even get a job; thus their social relationships get broader. Moreover, since they have to do many things alone, it leads to a sense of self-efficacy and a positive sense of self and social maturity.

These results can be carefully considered by women’s supporting organizations and institutions such as the Welfare Organization and the Relief Committee (Committee Emdad) to use in their plans and interventions. Concerted efforts to promote female-headed households’ social position and lessen negative experiences, along with policy-making and intervention to reproduce positive experiences, can make the future of this group of women less challenging and could enhance their individual, family and social status.

### Limits and strengths

This research is one of the few studies that have been done qualitatively on the problems of women heads of households in Iran. Previous studies have viewed this problem from an economic point of view. They do not reflect the social and cultural problems of women. Therefore, this research may reveal many unknown aspects for researchers and social actors. Previous research has not looked at female-headed households as an opportunity so far, and this study for the first time this study reveals the opportunities created by taking on the head of the household role. Since one of the authors of this article had an experience of working with support centers for female-headed households, it was beneficial in advancing research and gaining the trust of participants.

One of the most critical limitations of this study was women’s reluctance to participate in the study, which was addressed by explaining the aims and the necessity of the research and the commitment not to disclose personal information. In some cases, women also demanded that the interviewer be a woman, which was eventually resolved by a trained female researcher. Since this research was conducted in an environment with different social and cultural structures with a small number of participants, it may not be easy to generalize the results. However, this problem is almost related to the philosophy of qualitative research and cannot be considered a significant weakness. Note, however, that the researchers attempted to address this deficiency to an acceptable level by adhering to the basic principles of qualitative research and how to report it.

## Conclusion

The results of the study showed that female-headed households confront many individuals, intra-family and social challenges that, in many cases, disrupt their personal and social life and endanger their health seriously. However, becoming a head of the household for women is not only associated with negative consequences but, in some cases, leads to positive outcomes such as positive self-concept and social maturity. Therefore, to promote the health and empowerment of female-headed households, multidimensional programs are needed that encompass all aspects of their health. Therefore, women can be prepared to adapt to new, multifaceted, and sometimes conflicting roles by training for needed technics. They must also be supported by more economic support so that the cycle of poverty and disability within their families stops, and their children can live better. From a societal point of view, it is also possible to increase the social status of female-headed households by providing appropriate education and helping to change gender stereotypes so that their role as head and manager of a family can be accepted readily in society in order not to face social problems such as the stigma of being unattended and social exclusion.

## Data Availability

The datasets used and/or analyzed during the current study are available from the corresponding author on reasonable request.
